# Editorial: Neurofinance

**DOI:** 10.3389/fnins.2021.629154

**Published:** 2021-11-15

**Authors:** GianMario Raggetti, Maria G. Ceravolo, Luca Passamonti, Bernd Weber

**Affiliations:** ^1^Department of Experimental and Clinical Medicine, Marche Polytechnic University, Ancona, Italy; ^2^BrainLine Association, Marche Polytechnic University, Ancona, Italy; ^3^Department of Clinical Neurosciences, University of Cambridge, Cambridge, United Kingdom; ^4^Institute of Experimental Epileptology and Cognition Research, University of Bonn, Bonn, Germany

**Keywords:** neurofinance, financial decision making, investor behaviour, financial market, neuroeconomics

Neuroeconomics has recently emerged as a new interdisciplinary scientific area aimed at examining the role of the brain that, as a physiological organ, influences, *automatically* and *unconsciousl*y, individual behaviour in financial decisions. Neurofinance investigates the brain's activity in financial agents' decision-making processes. The most salient stages of the decision-making process are the collection of visual and auditory stimuli while receiving information on investment options, the selection and classification of such stimuli, their processing, and the interpretation. These phases are influenced by genetic and personality traits, implicit (unaware) memory of experiences, and by risk, or uncertainty, or ambiguity perception related to the available financial information and the market context. The brain automatically and unconsciously does many things before a decision is made and agents are aware of it. Neurofinance combines financial theories with psychology and neuroscientific insights to explain, in an interdisciplinary way, the widespread irrationality in the behaviour of financial operators. The role of emotion, mental status, biases, stress, personality, gender, age, and experience is therefore detected and analysed using different non-invasive clinical tools (i.e., fMRI, TMS, EEG, heart rate measurement, skin conductance detection, eye-tracking, hormone level measurement, neurotransmitters, and genome maps). Neurofinance researchers collect neural signals to understand what happens in the *black box* during the financial decision-making process. Since 2005, the articles and scientific texts in Neurofinance have increased annually. In this Neurofinance Research Topic, the first type of article includes the works of: Ceravolo et al. *Attention Allocation to Financial Information: The Role of Colour and Impulsivity Personality Trait*. The authors use eye-tracking technology, with an ecological protocol, to examine the patterns of eye movements in the early phases of information acquisition, during the reading of the KIID (a standardised document that provides essential information for investment decisions), disentangling the independent role of colour and impulsivity at modulating attention distribution. They observed that the increased attention induced by colour compensates for individual impulsivity, providing relevant insights for regulators who aim at increasing transparency and investors' protection. Jin et al., in their article ‘*Stimulating the Dorsolateral PreFrontal Cortex (DLPFC) decreases the Asset Bubbles*’ discuss the causal relationship and transmission mechanism of the DLPFC and asset bubble and examine different forecasting rules across individuals. The authors inferred that cognitive ability might be an important transmission mechanism in reducing asset bubbles. Wang et al., in their article ‘*Anodal tDCS over the right temporoparietal junction lowers overbidding in contests*’, using tDCS to modulate the activation of the rTPJ, investigate the neural underpinnings of overbidding—that is a commonly observed behaviour in competitive social interactions such as contests or auctions—and how the rTPJ impacts bidding behaviour. Findings suggest that the activation of the rTPJ in contests affects overbidding and bidding strategy, and further confirm that, in a competition context, the rTPJ is involved in the inference of mental states. In the second type of article, we consider the works of: Liao et al. *Exogenous Testosterone increases Decoy Effect in Healthy Males*. The authors studied the role played by testosterone in financial decisions and how it modulates decision-making when an asymmetrically dominated decoy option is introduced in a choice set. Results showed that participants in the testosterone group made fewer consistent choices and more target choices (i.e., decoy effect) than participants in the placebo group. These findings are interpreted in light of the dual-process theory based on existing evidence suggesting that testosterone promotes more intuitive and automatic judgments in human decision-making. Ramchandran et al., in their article ‘*The Role of Emotional vs. Cognitive Intelligence in Economic Decision-Making Amongst Older Adults*’ explore the links between emotions, bio-regulatory processes, and economic decision-making, which are well established in the context of age-related changes in fluid, real-time, decision competency. They explored this decision-making competency in the neurobiology of ageing by examining the neuroanatomical correlates of intelligence and decision-making, especially in regions that form a subset of the human mirror-neuron system. The data indicate that emotional intelligence plays a significant role in the economic decisions of older adults. McCormick et al., in their review article ‘*Neural Underpinning of Financial Decision Bias in Older Adults: Putative Theoretical Models and a Way to Reconcile them*’ focus on the topic that older adults face a perspective of reducing their economic wellbeing, autonomy, and happiness. Some factors such as increased medical expenses, reduced mobility, deficits in eyesight and hearing, and others lead them to retire, accepting a decreasing retirement income. They describe the processes of PFC decline, altered E-RP, and altered DMN in ageing, which, together, can affect financial decision-making. An alternative framework for understanding the neural network underpinnings of financial decision bias in older adults is proposed. Extracting *event-related potentials* (ERPs) from electroencephalographic data of a sample of top executives in real enterprises, Zang et al., in their paper ‘*The Neural Basis of Herding: Decision in Enterprise Clustering: An Event-relatEd Potential Study*’ examined the neural basis and processing of herding decisions in people's daily lives, showing the existence of herding tendency in business decisions. ERP results indicated that anti-conformity choices induced a larger amplitude than herding choices, demonstrating that participants might experience larger perceived risk and more decision conflict when they processed anti-conformity choices. In their fMRI study, ‘*Cash, Card, Smartphone: The Neural Correlates of Payment Methods*’, Ceravolo et al. focus on information technology innovations that have pushed towards the irreversible digitalization of payments. The authors tried to understand if and how brain activity can be modulated by the method of payment (cash, card, and smartphone), the paid amount, and/or both. They observe a greater activity of areas processing the perceived utility of motor behaviour (e.g., the parietal cortex), and the individual emotional involvement (e.g., INS). The authors suggest that cash enhances the salience and negative affective valence of paying by cash. The future development of Neurofinance will not only concern how to improve knowledge of the neural aspects of a financial agent's decision-making, but also new research areas related to building bridges between neuroscience and applications in business, to increase the value added, transparency, particularly in the financial market, and higher effectiveness of financial investor protection rules.

## Author Contributions

All authors listed have made a substantial, direct and intellectual contribution to the work, and approved it for publication.

## Conflict of Interest

The authors declare that the research was conducted in the absence of any commercial or financial relationships that could be construed as a potential conflict of interest.

## Publisher's Note

All claims expressed in this article are solely those of the authors and do not necessarily represent those of their affiliated organizations, or those of the publisher, the editors and the reviewers. Any product that may be evaluated in this article, or claim that may be made by its manufacturer, is not guaranteed or endorsed by the publisher.

